# Supplementary material for: Drug-Disease Severity and Target-Disease Severity Interaction Networks in COVID-19 Patients
Source: Pharmaceutics. 2022 Aug 30;14(9):1828. doi: 10.3390/pharmaceutics14091828 (PMC9504398; doi:10.3390/pharmaceutics14091828)
Supplement: Supplementary file 1 [file pharmaceutics-14-01828-s001.zip › pharmaceutics-1750333-supplementary.pdf]

Supplements:

Drug-disease severity and target-disease severity interaction networks in COVID-19 patients

Table S1: Assignment of ATC codes to the drug groups and subgroups

Group	Subgroup	Included ATC codes
Analgesics	Antimigraine preparations	N02C
	Nonsteroidal anti-inflammatory drugs (NSAIDs)	M01A, C01EB16, R02AX02
	Opioids	N02A
	Other analgesics and antipyretics	N02B
Antacids	Antacids	A02A
Antiepileptics	Antiepileptics	N03
Antihemorrhagics	Antifibrinolytics	B02A
	Vitamin K and other hemostatics	B02B
Antihistamines	Antihistamines	R06A
Antihyperglycemics	Alpha glucosidase inhibitors	A10BF, A10BD17
	Biguanides	A10BA, A10BD01, A10BD02, A10BD03, A10BD05, A10BD07, A10BD08, A10BD10, A10BD11, A10BD13, A10BD14, A10BD15, A10BD16, A10BD17, A10BD18, A10BD20, A10BD22, A10BD23, A10BD25, A10BD26
	Dipeptidyl peptidase 4 (DPP-4) inhibitors	A10BH, A10BD19, A10BD21, A10BD24, A10BD25, A10BD26, A10BD07, A10BD08, A10BD09, A10BD10, A10BD11, A10BD12, A10BD13, A10BD18, A10BD22
	Glucagon like peptide 1 (GLP.-1) analogues	A10BJ
	Other blood glucose lowering drugs, excl. insulins	A10BX
	Sodium glucose co-transporter 2 (SGLT 2) inhibitors	A10BK, A10BD14, A10BD15, A10BD16, A10BD19, A10BD20, A10BD21, A10BD23, A10BD24, A10BD25, A10BD19, A10BD20, A10BD21

Group	Subgroup	Included ATC codes
	Sulfonylureas	A10BB, A10BD01, A10BD02, A10BD04, A10BD06,A10BD14
	Thiazolidinediones	A10BG, A10BD09, A10BD12, A10BD03, A10BD04, A10BD05, A10BD06, A10BD26
Anti-infectives	Antibiotics	J01
	Antifungals	D01B
	Antivirals	J05
Antiobesity	Antiobesity	A08
Anti-Parkinson drugs	Anticholinergic agents	N04A
	Dopaminergic agents	N04B
	Other anti-Parkinson drugs	N04C
Anticoagulants	Direct factor Xa inhibitors	B01AF
	Vitamin K antagonists	B01AA
	Heparin	B01AB
	Direct thrombin inhibitors	B01AE
	Other antithrombotic agents	B01AX
Antiplatelet	Platelet aggregation inhibitors	B01AC
	Acetylsalicylic acid	B01AC06, N02BA01, M01BA03, N02BA51, B01AC56, C10BX08, C10BX12, C10BX06, C07FX04, N02AJ07, N02AJ02, C07FX03, N02AJ18, C10BX02, C10BX05, C10BX01, C10BX04, C07FX02
Calcium homeostasis	Parathyroid hormones and analogues	H05A
	Anti-parathyroid agents	H05B
Cardiovascular drugs	Antihypertensives	C02
	Beta blockers	C07, C09BX, C09DX
	Calcium channel blockers	C08, C09BB, C07FB, C09DB, C10BX03, C10BX09, C10BX14, C10BX18, C10BX07, C10BX11, C10BX09
	Cardiac therapy	C01
Cold drugs	Expectorants	R05C
	Cough suppressants	R05D
	Expectorants and cough suppressants	R05F

Group	Subgroup	Included ATC codes
	Cold other	R05X
Corticosteroids	Corticosteroids, inhalative	R03AK07, R03AK08, R03AK09, R03AK10, R03AK11, R03AK12, R03AK13, R03AK14, R03AL08, R03AL09, R03AL11, R03AL12, R03BA
	Corticosteroids systemic	H02B, H02AB, M01BA, A07EA, A01AC
Diuretics	(in the ATC not further specified diuretics, therefore not part of any subgroup)	C07C, C08G, C09BA, C09DA, C02L
	Loop diuretics (high ceiling diuretics)	C03C, C03EB
	Low-ceiling diuretics	C03B
	Potassium-sparing diuretics	C03DA, C03DB, C03E
	Thiazids	C03A, C07B, C07D, C03EA01, C03EA02, C03EA04, C03EA06, C03EA07, C03EA13, C10BX13, C10BX14
Immunglobolins	Immunglobolins	J06B
Immunosuppressants	Immunosuppressants	L04
Muscle relaxants	Muscle relaxants, peripherally acting agents	M03A
	Muscle relaxants, centrally acting agents	M03B
	Muscle relaxants, directly acting agents	M03C
Obstructive airway diseases	Adrenergic, inhalants	R03A
	Other drugs for obstructive airway diseases, inhalants	R03B
	Adrenergic for systemic use	R03C
	Other systemic drugs for obstructive airway diseases	R03D
Insulins	Insulins	A10A
Pancreatic hormone	Pancreatic hormone	H04
Drugs for peptic ulcer and gastro-esophageal reflux disease (GORD)	H2-receptor antagonists	A02BA
	Prostaglandins	A02BB
	Proton pump inhibitors (PPIs)	A02BC, B01AC56
	Combinations for eradication of Helicobacter pylori	A02BD
	Other drugs for peptic ulcer and gastro-oesophageal reflux disease (GORD)	A02BX

Group	Subgroup	Included ATC codes
Pituitary and hypothalamic hormones and analogues	Anterior pituitary lobe hormones and analogues	H01A
	Posterior pituitary lobe hormones	H01B
	Hypothalamic hormones	H01C
Psychoanaleptics	Antidepressants	N06A, N06CA
	Psychostimulants, agents used for ADHD and nootropics	N06B, N06CB
	Anti-dementia drugs	N06D
Psycholeptics	Antipsychotics	N05A
	Anxiolytics	N05B
	Sedatives	N05C
RAAS system agents	ACE inhibitors	C09A, C09B, C10BX04, C10BX06,C10BX07, C10BX17, C10BX18, C10BX12, C10BX13, C10BX15, C10BX14
	Renin inhibitors	C09X, C09DX
	Sartans (angiotensin II receptor blockers)	C09C, C09D, C10BX16, C10BX10
Respiratory other	Respiratory other	R07
Statins	Statins	C10AA, C10BA, C10BX
Supplements	Melatonin	N05CH01
	Vitamin A	A11CA, A11CB
	Vitamin B	A11D, A11E, A11HA02, A11HA04
	Vitamin C	A11G, A11EB
	Vitamin D	A11CC, A12AX, A11CB
	Zinc	A12CB, A16AX05, B05XA12, B05XA18
Thyroid therapy	Thyroid preparations	H03A
	Antithyroid preparations	H03B
	Iodine therapy	H03C

Table S2: Important network metrics of DDSI and TDSI network

Main node (hub): node with the highest weight, i.e. the largest share of patients taking this drug, weight in % is indicated in the brackets;
Main edge: edge with the highest weight, i.e. the largest share of patients taking this drug combination, weight in % is indicated in the brackets;
Diameter: maximum distance between any two nodes; or: the longest shortest path;
Node betweenness centrality: betweenness, indicating how often a node lies on the shortest path between two other nodes, nodes with the highest differences in betweenness between the severity cohorts are shown

	**Non-severe**	**Severe**
**Main node (hub) (weight in %)**		
Anatomical/ pharmacological groups	Blood and blood forming organs (85.6)	Blood and blood forming organs (94.8)
Drug group	Analgesics (59.7)	Analgesics (60.9)
Drug subgroup	Other analgesics and antipyretics (56.4)	Other analgesics and antipyretics (54.8)
Molecular target	Prostaglandin G/H synthase 1 (68.9)	Prostaglandin G/H synthase 1 (75.0)
**Main edge (weight in %)**		
Anatomical/ pharmacological groups	Blood and blood forming organs – nervous system (63.1)	Blood and blood forming organs – nervous system (62.6)
Drug group	Analgesics – anticoagulants (33.1)	Analgesics – anticoagulants (34.8)
Drug subgroup	*Other analgesics and antipyretics – heparin (24.4)*	*Other analgesics and antipyretics – antibiotics (28.7)*
Molecular target	Prostaglandin G/H synthase 1 – Prostaglandin G/H synthase 2 (65.4)	Prostaglandin G/H synthase 1 – Prostaglandin G/H synthase 2 (67.3)
**Diameter**		
Anatomical/ pharmacological groups	2.82	5.22
Drug group	1.55	3.48
Drug subgroup	1.04	2.61
Molecular target	1.3	2.88
**Node betweenness centrality**		
Anatomical/pharmacological groups		
Antiparasitic products	70	25.9
Dermatologicals	0	20.1
Musculo-skeletal system	0	15.5
Systemic hormonal preparations	0	11.2

	**Non-severe**	**Severe**
Antineoplastic and immunomodulating agents	1	9.8
Genito urinary system and sex hormones	8	1.3
Sensory organs	0.7	3.9
Various	0	2.4
Drug group		
Calcium homeostasis	108.7	0
Muscle relaxants	80.1	0
Pituitary and hypothalamic hormones and analogues	52.3	91.2
Anti-infectives	29.3	0
Thyroid therapy	0	26.6
Anti-Parkinson drugs	3	28.7
Antihistamines	42.7	19
Diuretics	18	0
Antiplatelet	16.9	0
Supplements	13.9	0
Drug subgroups		
Adrenergics for systemic use	113.5	0
Antihypertensives	108	12.3
Anti-parathyroid agents	76.8	0
Other analgesics and antipyretics	79.2	22.8
Immunosuppressants	74.7	24.4
Vitamin B	75.7	25.9
Biguanides	14.3	62.7
Glucagon like peptide 1 (GLP-1) analogues	53.2	6.4
Calcium channel blockers	2.7	44.7
Other blood glucose lowering drugs, excl. insulins	42	0
Molecular targets		
Annexin A1	1687.4	235.9
DNA	1043.8	0
Glucagon-like peptide 1 receptor	1110.8	113.3
P-glycoprotein 1	1191.4	224.8
Reverse transcriptase/RNaseH	1145.9	195.5
Solute carrier family 12 member 1	1225.5	320
Coagulation factor X	1701.3	848.2
Transcription factor AP-1	853.3	2.9

	Non-severe	Severe
Dipeptidyl peptidase 4	958	128.4
Antithrombin-III	1405.8	638.2

Table S3: Disease specific differences in the nodes of the DDSI network

Only nodes with significant differences are shown, as confirmed by Chi square test.

Heart: patients suffering from cardiac conditions (chronic heart failure, atrial fibrillation, coronary heart dis-ease and/or coronary sclerosis)
Diabetes: patients suffering from pre-diabetes, diabetes type 1 or diabetes type 2

**Drug groups**	**Non-severe COVID-19 [%]**	**Severe COVID-19 [%]**	**p value**
Heart			
Anti-hyperglycemics	*27.78*	12.07	0.03
Diabetes and heart			
Anti-hyperglycemics	*71.43*	33.33	0.007
**Drug subgroups**			
Heart			
Dipeptidyl peptidase 4 inhibitors	*12.7*	1.72	0.034
Diabetes and heart			
Dipeptidyl peptidase 4 inhibitors	*32.65*	4.76	0.029

Table S4: Disease specific differences in the edges of the DDSI network

Only edges with significant differences are shown, as confirmed by Chi square test.

Heart: patients suffering from cardiac conditions (chronic heart failure, atrial fibrillation, coronary heart dis-ease and/or coronary sclerosis)
Diabetes: patients suffering from pre-diabetes, diabetes type 1 or diabetes type 2
Arterial hypertension: patients with arterial hypertension

**Drug group combinations**		**Non-severe COVID-19 [%]**	**Severe COVID-19 [%]**	**p value**
Diabetes				
Anti-hyperglycemics	Anticoagulants	*46.67*	23.53	**0.029**
Heart				
Anti-hyperglycemics	Anticoagulants	*23.81*	6.9	**0.011**
Diabetes and heart				
Anti-hyperglycemics	Anticoagulants	*61.22*	19.05	**0.003**
	Statins	*53.06*	23.81	**0.046**
Arterial hypertension				
Antiplatelet agents	Cardiovascular drugs	22.53	*37.5*	**0.030**

**Drug group combinations**		**Non-severe COVID-19 [%]**	**Severe COVID-19 [%]**	**p value**
	GORD all	12.64	*25*	**0.033**
	Anti-infectives	11.54	*23.44*	**0.035**
Insulins	Psychoanaleptics	1.65	*7.81*	**0.048**
**Drug subgroup combinations**				
Diabetes				
Antipsychotics	Sartans	1.9	*11.76*	**0.048**
Heart				
Antipsychotics	Antiepileptics	0.79	*10.34*	**0.006**
	Proton pump inhibitors	3.97	*15.52*	**0.014**
	Other analgesics	7.94	*20.69*	**0.026**
	Loop diuretics	3.17	*12.07*	**0.042**
Arterial hypertension				
Antipsychotics	Opioids	1.65	*10.94*	**0.004**
	Adrenergic inhalatives	0.55	*7.81*	**0.006**
	Loop diuretics	2.75	*12.5*	**0.007**
	Proton pump inhibitors	2.75	*12.5*	**0.007**
	Antiepileptics	1.1	*7.81*	**0.019**
	ACE inhibitors	1.65	*7.81*	**0.048**
	Beta blockers	4.4	*12.5*	**0.049**
Acetylsalicylic acid	Proton pump inhibitors	10.44	*23.44*	**0.017**
	Beta blockers	14.84	*28.12*	**0.029**
	Potassium sparing diuretics	1.65	*7.81*	**0.048**
Loop diuretics	Heparin	7.14	*18.75*	**0.016**
	Opioids	4.4	*14.06*	**0.019**
Platelet inhibitors	Proton pump inhibitors	12.09	*25*	**0.024**
	Beta blockers	15.93	*29.69*	**0.027**
Insulins	Antidepressants	1.65	*7.81*	**0.048**

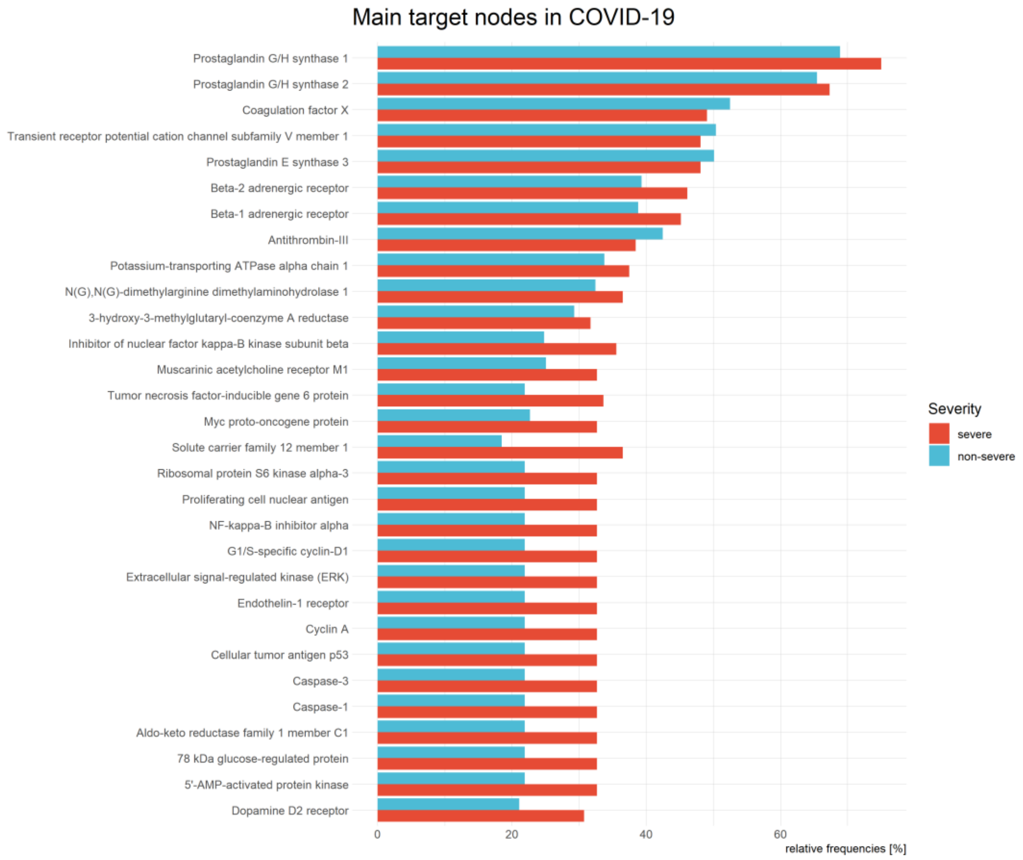

Figure S1: Main target nodes

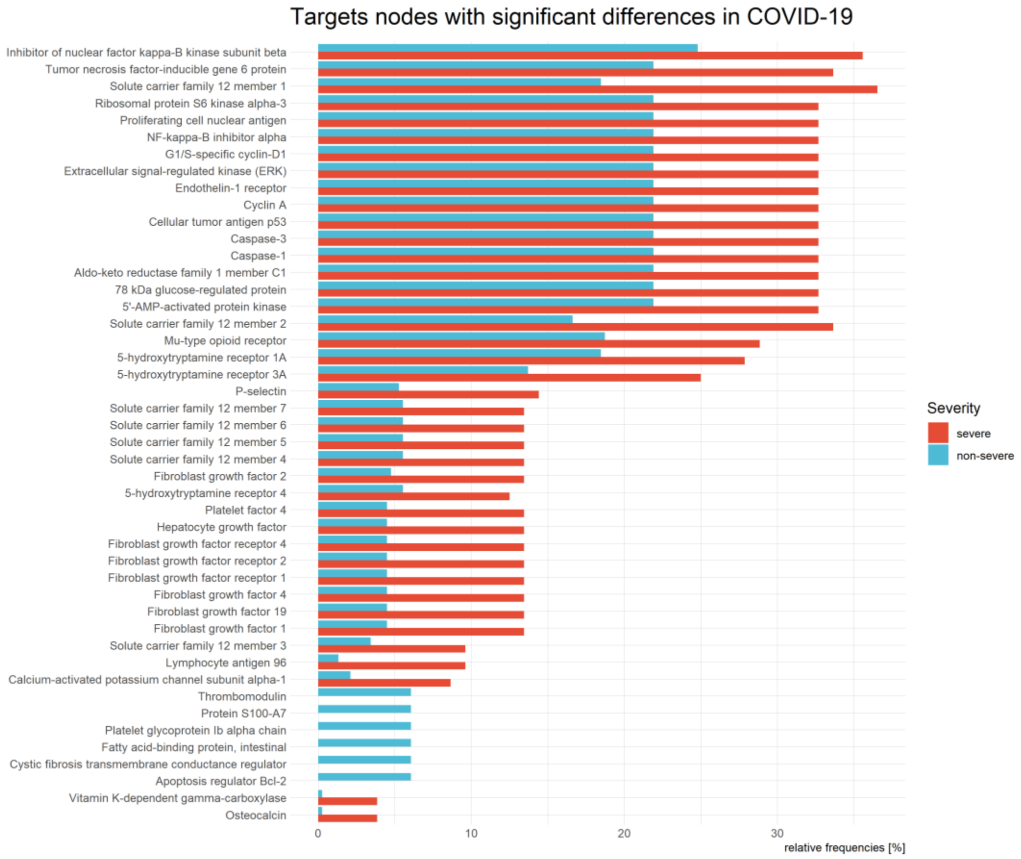

Figure S2: Target nodes with significant differences (p<0.001)

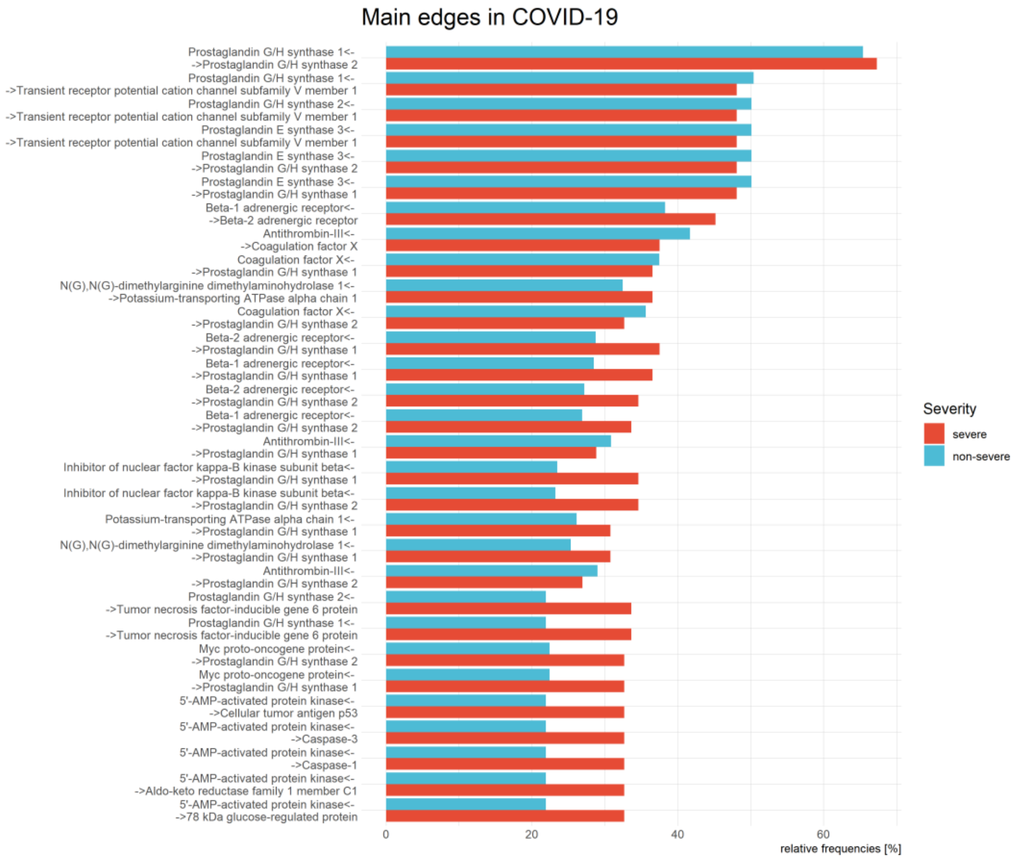

Figure S3: Main target edges

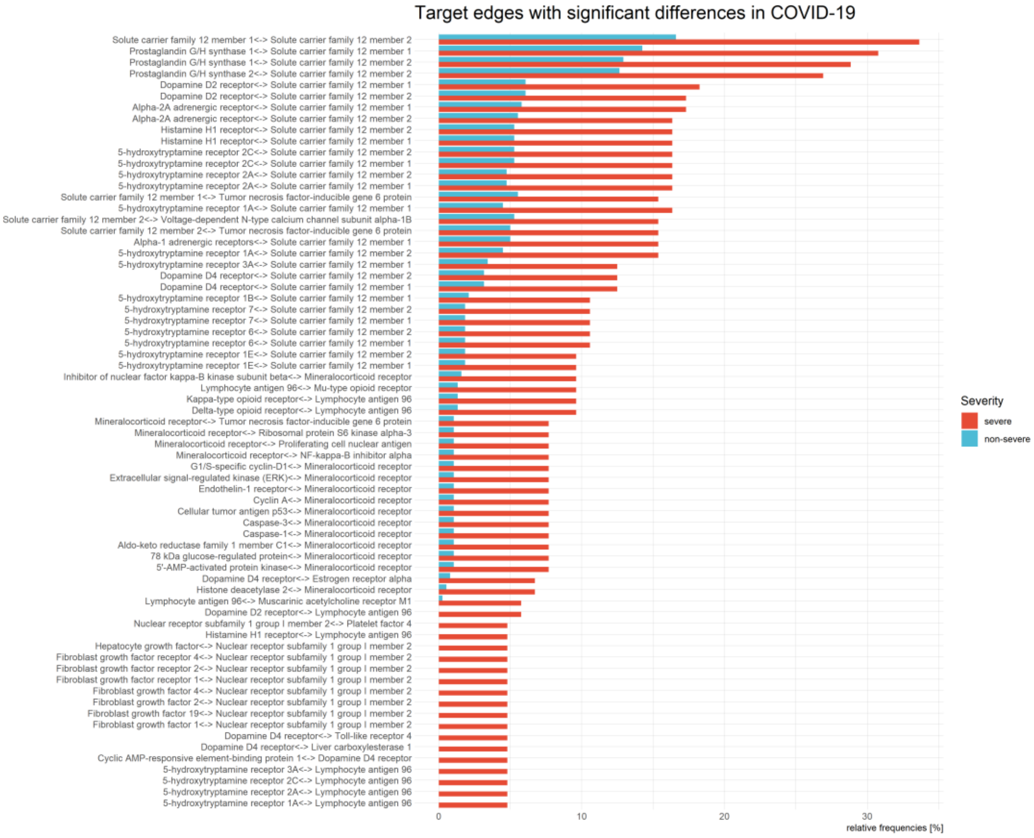

Figure S4: Target edges with highly significant differences (p<0.001)
